# Some Pieces Are Missing: Implicature Production in Children

**DOI:** 10.3389/fpsyg.2018.01928

**Published:** 2018-10-24

**Authors:** Sarah F. V. Eiteljoerge, Nausicaa Pouscoulous, Elena V. M. Lieven

**Affiliations:** ^1^Psychology of Language, University of Göttingen, Göttingen, Germany; ^2^Leibniz ScienceCampus Primate Cognition, Göttingen, Germany; ^3^Psychology and Language Sciences, University College London, London, United Kingdom; ^4^ESRC International Centre for Language and Communicative Development, School of Health Sciences, University of Manchester, Manchester, United Kingdom

**Keywords:** scalar implicatures, production, *some*, corpora, pragmatic development, language acquisition

## Abstract

Until at least 4 years of age, children, unlike adults, interpret *some* as compatible with *all*. The inability to draw the pragmatic inference leading to interpret *some* as *not all*, could be taken to indicate a delay in pragmatic abilities, despite evidence of other early pragmatic skills. However, little is known about how the production of these implicature develops. We conducted a corpus study on early production and perception of the scalar term *some* in British English. Children's utterances containing *some* were extracted from the dense corpora of five children aged 2;00 to 5;01 (*N* = 5,276), and analysed alongside a portion of their caregivers' utterances with *some* (*N* = 9,030). These were coded into structural and contextual categories allowing for judgments on the probability of a scalar implicature being intended. The findings indicate that children begin producing and interpreting implicatures in a pragmatic way during their third year of life, shortly after they first produce *some*. Their production of *some* implicatures is low but matches their parents' input in frequency. Interestingly, the mothers' production of implicatures also increases as a function of the children's age. The data suggest that as soon as they acquire *some*, children are fully competent in its production and mirror adult production. The contrast between the very early implicature production we find and the relatively late implicature comprehension established in the literature calls for an explanation; possibly in terms of the processing cost of implicature derivation. Additionally, *some* is multifaceted, and thus, implicatures are infrequent, and structurally and contextually constrained in both populations.

## 1. Introduction

A lot of information conveyed in conversation is not communicated explicitly, but implicitly; it is left for the audience to infer. For instance, if a student says she “read some of the papers assigned,” the listener may infer that she has not read all of them even though this was not been stated. Deriving the implicit interpretation of an utterance seems challenging for young children (Noveck, 2001; Papafragou and Musolino, 2003). Most work on how children come to grips with implicit meaning was carried out on scalar terms such as *some*. These expressions are part of a semantic informativeness scale (e.g., *some, most, all*) and the use of a weaker term on the scale (*some* or *most*) will often be taken to imply the negation of the stronger term (*all*) giving rise to a scalar implicature.

In experimental contexts, children, unlike adults, interpret *some* as compatible with *all*, and are not found to be adult-like until seven (Noveck, 2001; Papafragou and Musolino, 2003; Guasti et al., 2005; Huang and Snedeker, 2009b). While the age at which children draw scalar implicatures has been pushed down in some paradigms, they are still not found to interpret *some* in a pragmatic way until at least 4 years of age (Pouscoulous et al., 2007; Katsos and Bishop, 2011).

One of the keys to the enigma of scalar implicature development has to be production. Indeed, little is known about how the most popular scalar term, *some*, is produced by children. In the hope to shed light on implicature competence in early childhood we conducted a corpus study looking at the production of the quantifier *some* by five British English children aged two to five and their caregivers.

Most experimental work on children's understanding of implicit meaning has focused on children's interpretation of scalar implicatures. These occur when a speaker chooses to use a weaker expression (e.g., *some*) where she could just as easily have used a stronger one (i.e., *all*) and the hearer thereby understands that she has reasons not to use the stronger one—either because she did not have sufficient information or because she knew that it was inappropriate to use the stronger expression.

According to Grice's (1957; 1989) widely accepted model, implicatures—including scalar implicatures—are propositions that the speaker intends to communicate even though she does not express them explicitly. Hearers can infer the intended implicature by assuming that the speaker is cooperative and that she tries, as much as possible, to follow the conversational maxims of quantity, relevance, truth, and manner. In the case of scalar expressions such as *some* the hearer assumes that the speaker abides by the first sub-maxim of quantity (“Make your contribution as informative as is required”), at least so long as she can honour the second sub-maxim of quality, as well (“Do not say that for which you lack adequate evidence”). Therefore, in the example above, the hearer can infer that the speaker intends to convey the upper-bounded reading of *some* (not *all*) either because she does not know if the student read all the papers, or because she knows that the student did not read all of them. Depending on the context, scalar terms may therefore have two different interpretations, either a lower-bounded reading where *some* is compatible with *all* or an upper-bounded one, which excludes *all*. It is important to bear in mind that in real conversational uses a context might neither clearly prompt nor exclude a *some*-related implicature; in such contexts the relevance of the stronger alternative (*all*) may be uncertain and hearers' intuitions might diverge on whether a scalar implicature was intended by the speaker.

Scalar implicatures are particularly interesting for two reasons. First, they have stirred up a lot of theoretical controversy in recent years (for a review, see Geurts, 2010). It is hotly debated whether these implicatures are an output of grammar (Chierchia et al., 2012) or of fully-fledge pragmatic inferences. Amongst the defenders of the latter position, some view them as regular implicatures (“particularised” implicatures, in Gricean terms), which are derived only when prompted by the context (Noveck and Sperber, 2007; Geurts, 2010), while others argue they are “generalised” implicatures—i.e., they arise unless the context blocks them (Horn, 1989) or even by default (Levinson, 2000). Second, scalar implicatures often arise from the use of specific terms such as *some*, which makes them much easier to use in experimental settings. And, indeed, fueled by the theoretical debates, scalars have given rise to an important body of adult empirical work (for a review, see Breheny, forthcoming). The assumption behind much work on pragmatic development is that the findings on scalar implicatures can be generalised to other types of implicit meaning. Most studies on scalar expressions focus on the quantifiers *some*. In practice, this means our knowledge on children and implicatures is largely based on their understanding of *some* (for other implicatures, see Noveck et al., 2009; Schulze et al., 2013; Wilson, 2017).

Noveck (2001) conducted the first systematic experiments on children treatment of scalar expressions. He asked 8- to 10-year-olds to assess sentences of the form “Some giraffes have long necks,” which are logically true, but pragmatically underinformative, since “all giraffes have long necks.” Most children accepted the pragmatically underinformative utterances as true (at rates of 89%), while adults tended to reject them as false (41% accepted these as true). Unlike adults, children accept (rather than reject) utterances expressed with relatively weak terms when a stronger one is called for, and thus appear to be more literal than adults. These results were supported at the time by classic studies that inadvertently included scalar expressions (Paris, 1973; Smith, 1980; Braine and Rumain, 1981). Since then, several studies further demonstrated the phenomenon using a range of experimental methods (Papafragou and Musolino, 2003; Feeney et al., 2004; Guasti et al., 2005; Huang and Snedeker, 2009b). The effect seems to hold cross-linguistically with quantifiers (Katsos et al., 2016) and can be generalised to other scalar expressions; it has been found with 5-year-olds with *or* (not *and*) (Chierchia, 2004), *might* (not *must*) (Noveck, 2001), *start* (not *finish*) as well as numerals (Papafragou and Musolino, 2003). In all these experiments, the great majority of children accepted the weaker term as compatible with a stronger one, whereas adults would either consider them to be incompatible or at the very least be equivocal. Taken together, these findings might suggest that young children are unable to derive pragmatic inferences prompted by scalar expressions (for reviews on developmental findings on scalars, see Siegal and Surian, 2004; Pouscoulous and Noveck, 2009; Katsos, 2014; Papafragou and Skordos, 2016).

Children's performance on these implicature comprehension tasks is not due to semantic shortcomings. Indeed, children acquire *some* and *all* at around age 2 in both comprehension (roughly 16 months) and production (at roughly 26 months, Fenson et al., 1994). Furthermore, control conditions on most of the experiments described above indicate that children have a good semantic grasp of the two quantifiers (although, for a more nuanced picture, see Barner et al., 2009; Horowitz et al., 2017). Yet, other factors may influence children's performance on linguistic tasks—and in particular their understanding of pragmatic phenomena. Most studies mentioned above involve some type of sentence verification task. Children have to judge the truth or, at least, the adequacy of an utterance, a task which taps into their metalinguistic abilities. These develop through childhood, and children have been shown to understand a pragmatic phenomenon at an earlier age when assessed on non-metalinguistic tasks (such as act-out tasks or picture selection tasks) than when their comprehension of the same phenomenon is established based on tasks involving metalinguistic skills (see, e.g., Bernicot et al., 2007). In some paradigms, children have been shown to derive scalar implicatures, suggesting their poor performance is not due to semantic or pragmatic inability. Indeed, 5-year-olds' performance improves when they are trained to detect pragmatic infelicities (Papafragou and Musolino, 2003; Guasti et al., 2005). Importantly, it also does when the implicature outcome is made more salient and relevant in context (Papafragou and Musolino, 2003; Guasti et al., 2005; Foppolo et al., 2012; Skordos and Papafragou, 2016). Even 4-year-olds have been shown to derive scalar implicatures in two paradigms. In one of them, the child's understanding was assessed using a ternary scale rather than a binary choice; children could reward the speaker's utterance with a small, medium, or large strawberry rather than decide they were right or wrong (Katsos and Bishop, 2011). In the other, a simplified act-out paradigm was designed aiming to reduce task cognitive load and the effort involved in deriving the scalar implicature (Pouscoulous et al., 2007). Thus, children have been found to compute scalar implicatures linked to *some* from 4 years onwards but not younger (Pouscoulous et al. 2007; Katsos and Bishop 2011; see Stiller et al., 2014, for comprehension of non-lexicalised scalar implicatures in 3-year-olds).

There is therefore still a gap between the moment children produce and understand *some* and the point where they have been shown to derive its upper-bounded reading in an experimental context. Four main accounts of this phenomenon have been put forward. According to Katsos and Bishop (2011), young children understand the scalar implicature linked to *some*, but they are pragmatically more tolerant than adults. This leads them to accept utterances with *some* in contexts where *all* would be more appropriate even though they perceive the term as under-informative. Skordos and Papafragou (2016) on the other hand, emphasise the importance of conversational relevance in accessing the stronger alternative (*all*), and thus deriving the scalar implicature. Specifically, they maintain that children's ability to consider the stronger alternative depends fundamentally on how relevant this alternative is in context. When the lexical alternative is explicitly present or when it is simply contextually relevant, children consider it and infer the scalar implicature. A third strand has argued that the processing cost of implicatures is too high for young children; while they have the ability to understand scalar implicatures, they often lack the resources to make a relatively effortful inference (Reinhart, 2004; Pouscoulous et al., 2007). Indeed, evidence suggests that even for adults, scalar implicatures can be cognitively taxing (Noveck and Posada, 2003; Bott and Noveck, 2004; Breheny et al., 2006; De Neys and Schaeken, 2007). Finally, lexicalist accounts claim that while young children know the meaning of quantifiers such as *some* and *all*, they have not yet acquired the overarching informativeness scale. This prevents them from comparing *some* to *all*, and thus, from deriving the scalar implicature (Barner et al., 2010, 2011; Hochstein et al., 2014). It is worth noting that these accounts are not necessarily mutually exclusive. The first three, in particular, are sometimes presented by their supporters as potentially complementary (Katsos, 2014; Papafragou and Skordos, 2016). The debate to establish the best account of children's early difficulties with scalar implicatures is still very much raging. Yet despite our knowledge of implicature acquisition being largely based on children's understanding of *some*, we know very little about its production by children—and only slightly more for adults.

A single study has looked at scalar implicature production in children. Katsos and Smith (2010) investigated how 7-year-olds fare with scalar implicatures from a speaker's as well as a hearer's perspective. In addition to a usual binary truth value judgment task, children were asked to provide descriptions themselves. While the 7-year-olds' performance on the sentence verification task resembles what was found in other studies, they produced informative sentences at very high rate. These findings could be taken to point toward a speaker/comprehender asymmetry—where children find production easier than comprehension—as is sometimes alluded to for other pragmatic phenomena (e.g., informativeness, Davies and Katsos 2010, and presuppositions, Berger and Höhle, 2012). Importantly, the authors do not attribute this apparent comprehension-production asymmetry to a lack of pragmatic competence, but to a different metalinguistic attitude in children when they have to judge utterances.

The ideal way to investigate the production of *some* is to study corpora of real use in addition to experimental methods. Three corpus studies have looked at adult production of *some*. The first is a small scale study in Huang and Snedeker (2009b), where they extracted 50 random instances of *some* from the British National Corpus and analysed them depending on whether they referred to a subset or not. More convincingly, Degen (2015) extracted 1748 occurrences of *some*-NPs from a telephone dialogue corpus. She excluded 359 *some*-NPs headed by singular count nouns and 26 cases where the NP consisted only of *some*. The remaining 1363 *some* instances were used in a web-based study. Participants recruited on Amazons Mechanical Turk were asked to judge the probability of an implicature being intended by assessing the similarity on a 7-point-Likert scale between the original *some* utterance and an “implicature paraphrase” resulting from inserting *but not all* after *some*—e.g., “I like to read some of the philosophy stuff” and “I like to read some, but not all, of the philosophy stuff.” Sun (2017) uses a very similar procedure to get implicature plausibility rates for several triggers extracted from twitter, including 200 instances of *some*. These studies were designed to test what Degen calls the “Frequency Assumption”; an implicit assumption found in much of the theoretical and empirical literature on scalars that lexicalised scalar terms, such as *some*, will more often than not give rise to implicatures. The findings show that the upper-bound reading of *some* is found in naturally occurring speech, but is not prevalent; a conclusion with important (negative) consequences for theories relying on a dominant upper-bound interpretation of scalar terms, such as the defaultism of Levinson (2000) or syntax-based approaches (Chierchia, 2006; Chierchia et al., 2012). These results also have implications for children's acquisition. Indeed, a low implicature rate in adult speech might account, in part, for their difficulties with the lower-bound interpretation of *some*.

At this juncture of our understanding of scalar implicature and its development, a study of naturalistic child and parent production seems essential. Such data are very difficult to get in experimental settings, particularly for children, and a child corpus analysis seems a more convincing way forward. Yet, while focusing on a corpus reflecting children's natural spontaneous speech, as well as their environment, comes with a host of advantages, it brings its own issues, too. How are we to assess the speaker's intention to produce an implicature? Degen (2015) solves this impasse by postulating that in communication, hearer's recognition of speaker's intention is, overall, a fair approximation of the speaker's intention: the audience's intuitions about implicatures correspond by and large to the speaker's intention to produce them. Unfortunately, when looking at younger children's production we cannot rely on implicature plausibility ratings from untrained Mechanical Turk participants. But, we can code for the plausibility of an implicature being intended by the use of *some*, based on the context of utterance and tests such as whether it refers to a subset (Huang and Snedeker, 2009b) or the *not all* paraphrase (Degen, 2015).

In the following, we therefore present a corpus study on young children's production of *some*, adding a missing piece to the current literature and our understanding of early pragmatic abilities. Children's utterances containing *some* were extracted from dense corpora of five children aged 2;00 to 5;01 (*N* = 5,276), and analysed alongside an equivalent portion of their mothers' utterances with *some*. These were coded into structural and contextual categories allowing for judgments on the probability of a scalar implicature being intended (coding scheme partly based on Degen, 2015).

## 2. Data and methods

### 2.1. The corpus

We looked at the production of *some* in dense corpora of five British English speaking children aged 2;00 to 5;01. Three sets (Thomas, Fraser and Eleanor) are part of the CHILDES database (MacWhinney, 2000; Lieven et al., 2009), while two (Gina and Helen) were accessed with the kind permission of the Child Study Centre, University of Manchester (De Ruiter et al., 2017). All families were from the Greater Manchester area in the United Kingdom. For each child, the corpus included dense recordings of 5 hours per week for the first 6 weeks following each of their birthdays, as well as 5 hours within one week during each of the subsequent months of the year. The interactions between children and their parents (mostly their mothers, a father appears once) took place at home usually during play, reading, or snack time. The children were recorded from 2;00 to 3;01 years for Eleanor and Fraser, from 2;00 to 4;11 years for Thomas, and from 3;00 to 4;07 years for Gina and Helen.

### 2.2. Coding

Children's utterances containing *some* were extracted with three lines of context before and after each *some* occurrence (*N* = 5,276). For each child, data were organised into age windows of 3 months allowing for an analysis of individual developmental trajectories. To examine inputs in the early years, we extracted the mothers' first sentences with *some* in a number equivalent to their child's production (*N* = 5,430). To further investigate input development, we extracted another 300 *some* utterances produced by each of the mothers after their child's birthdays (*N* = 3,556; Total number of utterances coded for mothers = 9,030). For one mother, the recording stopped after 256 utterances after the child's last birthday, meaning that 300 utterances could not be reached. All 14,306 utterances were categorised following structural and contextual categories allowing for judgments on the probability of a scalar implicature.

All utterances were first coded following a structural grid, according to the type of syntactic structure the word *some* appeared in. Eleven structural categories were established: Seven were marked as *Included* and four as *Excluded*. Utterances falling under the *Included* categories were subsequently coded according to the contextual coding scheme while utterances falling within the *Excluded* categories could not be coded further due to missing or incomplete information (e.g., errors, ambiguities). In a second phase, the *Included* cases were coded according to their likelihood of carrying an implicature from *some* to *not all*. Four contextual categories were devised to reflect judgment on the probability of an implicature being intended: *Implicature Impossible, Implicature Implausible, Implicature Possible*, and *Implicature Plausible*. The coding scheme was adapted in part from Degen (2015), and was used equally for children and adult uses of *some*. The data and coding of the corpus reported in this paper are accessible to readers on the Open Science Framework database at osf.io/g6psr.

#### 2.2.1. Structural categories

All the extracted *some* utterances were coded as belonging to one of the mutually exclusive, structural categories outlined in Table 1. There are seven *Included* categories.

1. In the *Mass* category, *some* precedes a mass noun including object mass nouns (e.g., coffee and furniture).
2. The *Count as mass* category includes count nouns that appear in a mass noun-like structure (e.g., *Want some banana*).
3. The *Adjective* category includes *some* utterances headed by an adjectival noun (often colours, e.g., *Need some blue*).
4. Similarly, in the *Plural noun* category the phrase is headed by a plural noun (e.g., *some people*).
5. The category *Singular NP* covers utterances with a singular count noun. Although the structure is similar to the *Count as mass* category they differ in the quantity of the referent; in the *Singular NP* cases it only refers to one single entity and not to a mass (cf. “Some guy predicted the end of the world today,” Degen, 2015, p. 5, Ex. 12).
6. The *Plural NP* category includes cases where *some* is followed by a count noun in its plural form (e.g., I need *some* blocks).
7. Finally, the *Of XP* category covers prepositional phrases (e.g., I need *some* of these toys).

**Table 1:** Structural categories, their definition, and examples.

	**Category**	**Structure**	**Example**	
Included
		Mass	mass NP	Mummy want *some* tea. (E., 2;00)
			object mass NP	Get *some* fruit from there. (E., 2;11)
		Count as mass	sg count NP for quantity	I like *some* banana. (E., 2;00)
		Adjective	adjectival NP	I need *some* yellow. (E., 2;00)
		Plural noun	pl NP for pl quantity	*Some* people love Peppa Pig. (H., 3;00)
		Singular NP	sg count NP	*Some* little boy kissed a chair. (H., 4;01)
		Plural NP	pl count NP	I want *some* dinosaurs. (E., 2;01)
		Of XP	partitive preposition	Mum keeps *some* of these balls. (E., 3;01)
Excluded		Solitary some	no spelled-out NP	Po like *some*. (E., 2;00)
		More	might mean *more*	I need *some more*. (E., 2;00)
		Structure unclear	pl NP for sg quantity	Need *some* scissors. (E., 2;00)
			conjunctive NPs	I've got *some* fish and chips cook. (E., 2;08)
		Transcription	*some* replaced	I've got *some* ¡a triangle¿. (E., 2;00)
		unclear	incomplete phrase	Let's play *some* +…[+ IN] (E., 2;03)
			transcription failure	Mummy, let's go *some* paint xxx. (E., 2;00)
			unclear utterance	I can do *some* [=? the] shopping. (E., 2;05)

*sg, singular; pl, plural; NP, Noun Phrase (fully compatible with DP analysis)*.

There are four *Excluded* categories. Utterances falling in one of these categories were not analysed further.

1. In the *Solitary some* category, *some* is not followed by a noun.
2. The *More* category, includes utterances with *some more*. These seem to mean *more* in the context of language acquisition as children are often asking for *some more* of food for example. Although it could be argued that *more* is used here as a modification, an implicature is implausible in most such cases.
3. In the category *Structure unclear*, two different types of uses are pooled.
  - Plural nouns such as scissors and pants were excluded, because it could not be established whether the noun refers to a single quantity or to a mass.
  - *Some* introducing *conjunctive* phrases were also excluded due to the structural ambiguity. Indeed, it could not be established whether *some* should be linked to the first conjunct or the whole conjunctive phrase.
4. The category *Transcription unclear* also includes several cases.
  - When the sentence includes the word *some*, but is continued with a replacement, the word *some* is not used to quantify anymore (e.g., I want some, a bread).
  - Incomplete phrases were excluded when the referent for *some* was missing (e.g., I want some +IN). When the referent for *some* was uttered in the next line of the transcription, the utterance was included since the referent of the *some* phrase was readily available (e.g., “I want some +IN. some grapes”).
  - Partly unintelligible sentences (transcribed with *xxx*) were also excluded.
  - When the transcription left a doubt about *some* being uttered, the utterance was excluded as well.

All occurrences of *some* were also independently coded according to additional, non-mutually exclusive, structural categories which impact discourse accessibility and therefore the likelihood of an upper-bounded reading of *some*. In doing so, we followed the approach of Degen (2015), and collected data which could inform how structural linguistic elements may influence implicature probability. These categories also provide further dimensions on which to compare child and adult production. For example, it has been argued that the subject position tends to support implicature interpretation (Degen, 2015). Breheny et al. (2006) suggested that a scalar implicature is more likely when in focus as focus highlights relevant content. This would then underline the contrast between *some* and *all*. The same holds for phrases that are topicalised, as the topic position is often associated with focus which can support contrasting *some* with *not all* (e.g., Some of the grapes the girls ate). Third, we coded whether the phrase was modified. On the one hand, modification can increase the salience of a novel mention in a discourse (Degen, 2015). On the other hand, modification can also counteract implicature plausibility when a set (e.g., of blocks) is then already subsetted (e.g., blue) which reduces the salience of *some* (e.g., I need some blue blocks). Forth, we coded whether *Of XP* phrases were headed by a pronoun or demonstrative. As Degen (2015) notes, pronoun and demonstrative phrases with *some* are ungrammatical when used without the partitive (Example 39 on p. 22: “And some ^*^(of) them fizzled out,” Degen, 2015). Nonetheless, in her study, sentences with and without pronouns or demonstratives receive similarly high ratings.

#### 2.2.2. Contextual categories

Utterances falling in one of the *Included* categories (see Table 1) were then assigned to one of four, mutually exclusive, contextual categories, which reflect their likeliness to carry an implicature based on structure and the extracted context (± 3 utterances): *Implicature Impossible, Implicature Implausible, Implicature Possible*, and *Implicature Plausible* (see Table 2).

1. For utterances categorised as *Implicature Impossible*, no quantifiable set could be identified of which *some* could have been a subset. With no clear set in the discourse, the speaker cannot intend to refer to a subpart through a scalar implicature (“I need *some* help”). For instance, this category includes cases of spontaneously occurring natural phenomena (like trumps or clouds).
2. In utterances categorised as *Implicature Implausible*, a quantifiable set could be found, but the speaker was unlikely to be referring to it in this context. For instance, it would be possible in some contexts to use the sentence “We need to buy some batteries” to refer to a subset of batteries. Yet, in the corpus, the context suggested a more general meaning of getting batteries.
3. In the occurrences categorised as *Implicature Possible*, a quantifiable set could be identified and it was possible that the speaker was using the quantifier *some* to refer to a subset via an implicature, for instance in “I ate some biscuits”. Yet, the available context does not provide sufficient elements to disambiguate between the two readings roughly paraphrased as “I ate biscuits” and “I ate some, but not all biscuits”.
4. Finally, *Implicature Plausible* utterances involved a clearly identifiable set, which was relevant to the conversational exchange and to a subset the speaker seemed to be referring to. Thus, the speaker seemed to have used *some* intending the hearer to derive the scalar implicature and understand *not all*. For instance, when in the context of playing with jigsaw puzzles, a child utters “The puzzle is missing some pieces.” Even in such cases, there can be no guarantee that the speaker intended to convey an implicature, rather we establish that the utterance is highly compatible with an implicature interpretation.

**Table 2:** Contextual categories indicating implicature plausibility.

**Category**	**Description**	**Examples**
Implicature impossible	No available set	I did *some* trumps. (E., 2;00)
		Blowing *some* bubbles. (T., 3;01)
Implicature implausible	Set possible but not referred to	Squirrel wants *some* nuts.(E., 2;00)
Implicature possible	Maybe referring to subset of set	Po's got *some* biscuits in his house. (E., 2;00)
Implicature plausible	Referring to subset of present set	I lost *some* pieces. (F., 3;00)

As mentioned in the introduction, we had to assess the likelihood of the speaker intending to convey an implicature based on the hearer's understanding of this intention—more specifically, we have to rely on the coder's pragmatic inferences. Therefore, to avoid false positives and inflating the proportion of intended implicatures, the less implicature-compatible category was chosen when in doubt about the most appropriate contextual category for an utterance.

To correctly categorise all phrases, certain tests were applied. As seen above, to establish implicature plausibility, Degen (2015) used similarity ratings with paraphrases where *some* was replaced by *some but not all* (e.g., “I ate some biscuits” and “I ate some, but not all, biscuits”). We used the paraphrase test as a guideline: high similarity would correspond to a categorisation as *Implicature Possible* or even *Implicature Plausible* when the context strongly supported an implicature reading.

However, note that *all* is not necessarily the upper bound in all discourses as it can also be interpreted differently in certain pragmatic contexts. For example, when *all* is used to exaggerate, it can actually mean *some* or *most* (e.g., “She ate *all* the biscuits!” when meaning that this person did not leave many biscuits for the rest of the group. See also section 4).

Another paraphrase test we used as a guideline was the omission of the quantifier. When *some* can be left out [as in “I need (some) help” or “We need to buy (some) batteries”], it seems to be used as an indefinite marker and the occurrence would be categorised as *Implicature Impossible* or *Implicature Implausible*. To decide between these two categories, the content was taken into account. When no set could be defined (as in “help”), then the *Implicature Impossible* category was chosen. When a set could be identified, but was either non-quantificational or not the topic of discussion (e.g., an existing set of batteries in the store, but not relevant to the dialog) the utterance would fall into the *Implicature Implausible* category.

Context remained crucial to judgments about categorisation. Take a child saying “I want to eat some grapes,” for instance. It is possible that there is a set of grapes in the kitchen. In most cases, it would be unlikely that the child is referring to that set. The implicature would thus be deemed *Implausible*. On the other hand, if the mother just uttered “See, there are some grapes on the table, the rest is in the kitchen,” now the context establishes clear, relevant subsets and the implicature of the mother's utterance seems *Plausible*. The same holds if the child said “I want to eat some grapes. The others are for you,” thereby actively differentiating between subsets.

A second coder independently coded 1,730 out of the 14,306 utterances of the overall corpus data; roughly 20% of Included and 9.5% of Excluded utterances split proportionally across children and adults, which sums up to roughly 12% of the whole corpus. Interrater reliability for all utterances was at 85% indicating very high agreement overall (contextual categories: 81% and Cohens Kappa of 0.7; structural categories: 89% and Cohens Kappa of 0.87. Cohens Kappa was calculated using confusion matrices with the package caret in R; Kuhn, 2013, for the use of Cohen's Kappa to assess interrater reliability, see Landis and Koch, 1977; Viera and Garrett, 2005; Cameron-Faulkner et al., 2007; Spooren and Degand, 2010).

## 3. Results

### 3.1. Mothers' usage

Categorisation of the 5,687 utterances coded for the mothers can be seen in the Tables 3 and 4. Note that the number of appearances deviates from the extracted utterances as *some* could appear more than once in a sentence.

**Table 3:** Results for the structural categories of the mothers' data.

	**Category**	***N***	**%**	
Included
		Mass	1,614	28.38
		Count as mass noun	480	8.44
		Adjective	23	0.40
		Plural noun	110	1.93
		Singular NP	63	1.11
		Plural NP	1,605	28.22
		Of X	277	4.87
		Solitary some	456	8.02
		More	689	12.12
Excluded				
		Structure unclear	156	2.74
		Transcription unclear	214	3.76

*Percentages are in proportion to all 5,687 utterances. N of Included utterances: 4,172; N of Excluded utterances: 1,515*.

**Table 4:** Results for the contextual categories of the mothers' data.

**Category**	***N***	**%**
Implicature impossible	710	17.02
Implicature implausible	2,774	66.49
Implicature possible	420	10.07
Implicature plausible	268	6.42

*Percentages are in proportion to all 4,172 Included utterances*.

Regarding the structural categories, the categories *Mass* and *Plural NP* dominated. Adjectival phrases were rare. Exclusion was highest for the *More* category.

The *some* phrase appeared rarely in subject position (*N* = 63, 1.15%), and was almost never topicalised (*N* = 3, 0.07%), and therefore mostly realised in object position. A small part of utterances was modified pre- or post-phrasal (*N* = 450, 10.79%). Around a quarter of all *Of XP* utterances were headed by a pronoun or a demonstrative (*N* = 75, 27.08%).

**Figure 1:**
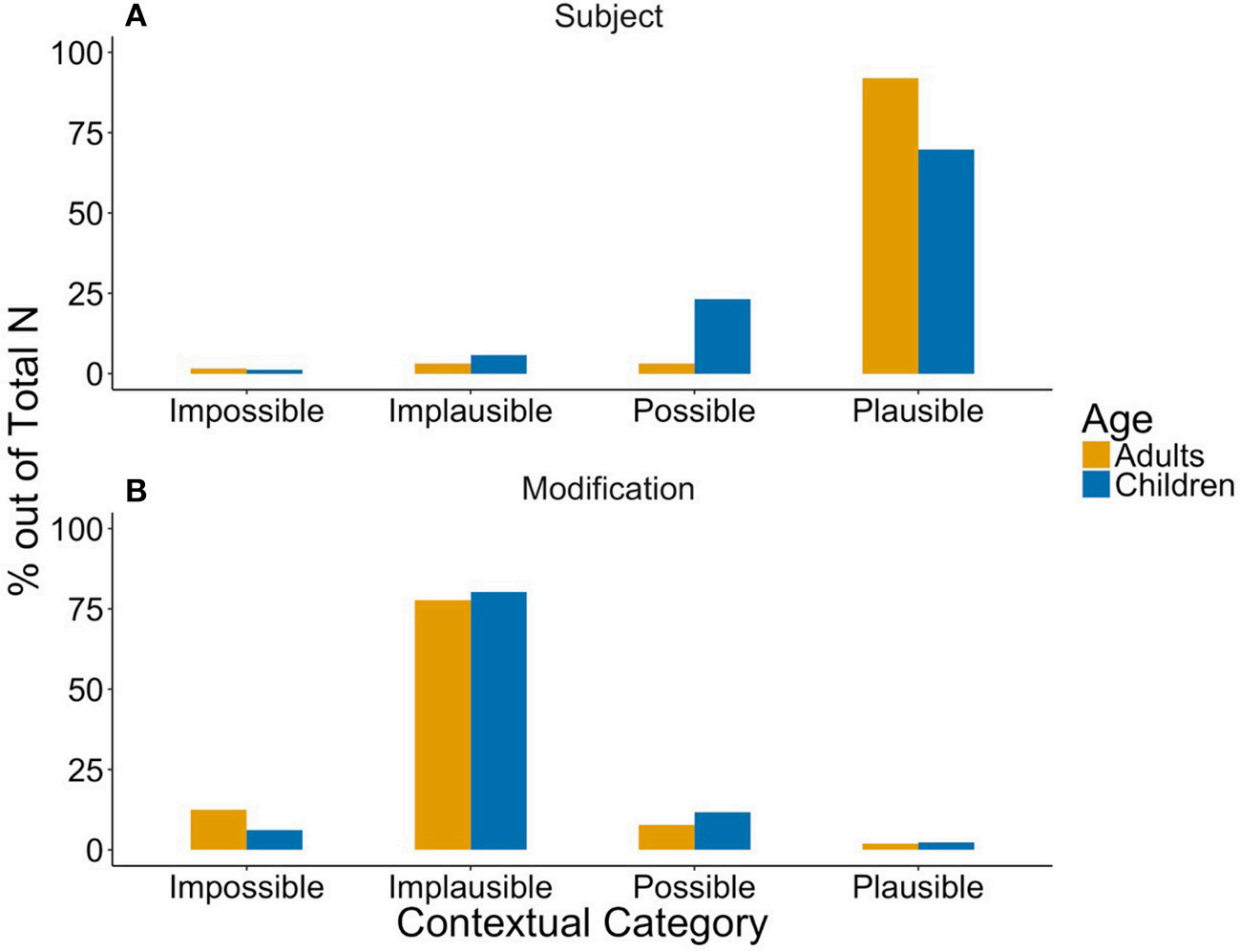
Structural influences on the implicature plausibility of *some* in **(A)** subject position (Adult *N* = 63, Child *N* = 86), and **(B)** *some* being modified (Adult *N* = 450, Child *N* = 214) in caregivers' (yellow) and children's (blue) production.

As in Degen (2015), structural properties seemed to relate to implicature plausibility as can be seen in Figure 1: While *some* in subject position supported an implicature reading (*Implicature Plausible* ratings), modifications were mostly found in the *Implicature Implausible* category.

In the contextual categories, *Implicature Plausible* utterances represented a small proportion of the *Included* set (6.42%), while most utterances were categorised as *Implicature Implausible* (66.49%).

Looking at the relation between structural and contextual categories we find more *Implicature Plausible* ratings in certain structural categories and close to none in others (see Table 5). For example, there were no *Implicature Plausible* cases amongst *Singular NP*. Cases of *Plural NP*, however, could belong to any of the four contextual categories. Furthermore, *Of XP* utterances were prone to be categorised as *Implicature Plausible* (*N* = 195, 70.4%). Thus, the partitive structure seems to support implicature interpretation. On the other hand, structures suggesting a singular quantity are difficult to combine with a partitive reading and are unlikely to give rise to an implicature reading. A structure such as the *Plural NP* category is more flexible; it allows for more variation in implicature readings, and its interpretation is therefore highly dependent on context.

**Table 5:** Contextual categorisation of the individual *Included* structural categories of the mothers' data.

**Category**	**Total *N***	**Impossible**		**Implausible**		**Possible**		**Plausible**	
		***N***	**%**	***N***	**%**	***N***	**%**	***N***	**%**
Mass	1,614	362	22.43	1,121	69.46	114	7.06	17	1.05
Count as mass noun	480	143	29.79	333	69.38	0	0	4	0.83
Adjective	23	0	0	23	100	0	0	0	0
Plural Noun	110	0	0	96	87.27	6	5.46	8	7.28
Singular NP	63	0	0	63	100	0	0	0	0
Plural NP	1605	205	12.77	1,138	70.9	218	13.58	44	2.74
Of XP	277	0	0	0	0	82	9.62	195	70.4

### 3.2. Children's usage

Table 6 provides the structural categorisation and Table 7 the contextual categorisation for all 5,310 *some* utterances of the children. Again, the number of appearances deviates from the extracted utterances as *some* could appear more than once in a sentence.

**Table 6:** Results for the structural categories of the children's data.

	**Category**	***N***	**%**
Included
	Mass	1,080	20.34
	Count as mass noun	279	5.25
	Some adjective	45	0.85
	Plural noun	75	1.41
	Singular NP	140	2.64
	Plural NP	1,078	20.3
	Of X	186	3.50
Excluded	Solitary some	754	14.20
	More	754	14.20
	Structure unclear	100	1.88
	Transcription unclear	819	15.42

*Percentages are in proportion to all 5,310 utterances. N of Included utterances: 2,883; N of Excluded utterances: 2,427*.

**Table 7:** Results for the contextual categories of the children's data, indicating plausibility of implicatures for included utterances.

**Category**	***N***	**%**
Implicature impossible	282	9.78
Implicature implausible	2,040	70.76
Implicature possible	322	11.17
Implicature plausible	239	8.29

*Percentages are in proportion to all 2,883 included utterances*.

Note that children, as their mothers, used *some* in several different structural forms and that, again as their mothers, there is a predominance of *Mass* and *Plural NP* usage. Adjectival phrases were rare for children, too. Exclusion was highest for the *Transcription unclear* category. Overall, more utterances had to be excluded than in the mothers' data suggesting that the data of the children were noisier, as would be expected considering their age.

As for their mothers, the *some* phrase appeared rarely in subject position (*N* = 86, 2.98%), and was never topicalised, and therefore mostly realised in object position. A small part of utterances was modified pre- or post-phrasal (N = 213, 7.39%). More than half of all *Of XP* utterances were headed by a pronoun or a demonstrative (*N* = 113, 60.75%) .

As in Degen (2015) and our adult data, structural properties seemed to relate to implicature plausibility as can be seen in Figure 1: While *some* in subject position supported an implicature reading (*Implicature Plausible* ratings), modifications were mostly found in the *Implicature Implausible* category.

Interestingly, in the children's contextual categorisation, implicature production can clearly be observed. A total of 19.46% of the *Included* cases were categorised as *Implicature Possible* or *Implicature Plausible*, despite the fact that the *Implicature Implausible* was still the most largely represented.

Here again, implicature plausibility diverged depending on the structural category as can be seen in Table 8. For example, *Singular NP* provided no *Implicature Plausible* cases, indicating that its structure is a cue against implicature plausibility as suggested by Degen (2015, p. 5). The *Plural NP* category however, provided utterances belonging to all four contextual categories. Thus, such a structure allows for more variation in implicature readings; whether it gives rise to an implicature interpretation or not is therefore highly dependent on context. As observed in the mothers' production, the *Of XP* category was prone to carry implicatures (*N* = 158, 84.95%). Therefore, the partitive structure supported implicature readings also in the children's data.

**Table 8:** Contextual categorisation of the individual *Included* structural categories of the children's data.

**Category**	**Total *N***	**Impossible**		**Implausible**		**Possible**		**Plausible**	
		***N***	**%**	***N***	**%**	***N***	**%**	***N***	**%**
Mass	1,080	135	12.5	829	76.76	103	9.54	13	1.2
Count as mass noun	279	54	19.36	225	80.65	0	0	0	0
Adjective	45	0	0	45	100	0	0	0	0
Plural Noun	75	0	0	30	40	17	22.67	28	37.34
Singular NP	140	0	0	140	100	0	0	0	0
Plural NP	1,078	93	8.63	769	71.34	176	16.33	40	3.71
Of XP	186	0	0	0	0	28	15.05	158	84.95

We also looked at children's individual production of *some* over time within the corpus to establish when different types of uses, as well as implicature production, first appear (see Table 9). The resulting developmental picture shows that children begin using *some* in its many forms during their third year of life. Importantly, this includes implicature production. Indeed, as can be seen in Table 9, the first *Implicature Plausible* instances of *some* produced by the three 2-year-olds appear 3 to 9 months after their first use of *some* in the corpus.

**Table 9:** Overall data of the individual children.

**Child**	**Recording**	**Total**	**Incl**	**Excl**	**1st *some***	**1st Category**	**1st implicature**
Eleanor	2;00 - 3;01	937	497	440	2;00;03	Mass	2;04;02
Fraser	2:00 - 3;01	627	359	268	2;00;28	Mass	2;03;06
Thomas	2;00 - 4;11	1770	906	864	2;00;13	Mass	2;09;11
Gina	3;00 - 4;07	971	504	467	3;00;01	Plural NP	3;00;04
Helen	3;00 - 5;01	1005	617	388	3;00;02	Plural NP	3;00;10

*Incl, N of utterances in included categories; Excl, N of utterances in excluded categories*.

Altogether, the findings indicate children's competence regarding different types of *some* including pragmatic production. To see whether their behaviour mirrors the input provided by their mothers, we next turn to the comparison of these results with child-directed speech.

### 3.3. Comparison of the children and their mothers

**Figure 2:**
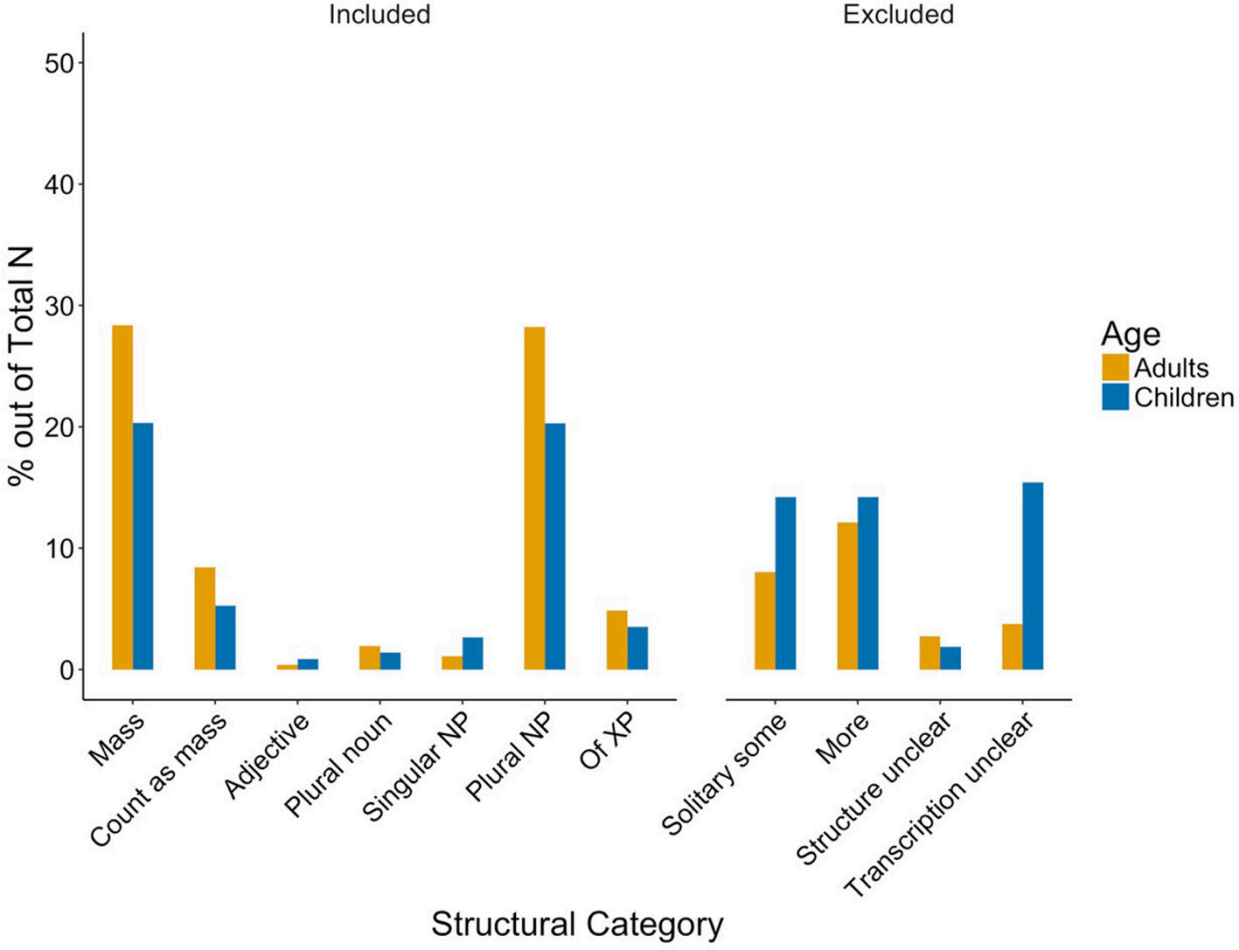
Structural categories in caregivers' (yellow) and children's (blue) production. Percentages are in proportion to all utterances per group.

**Figure 3:**
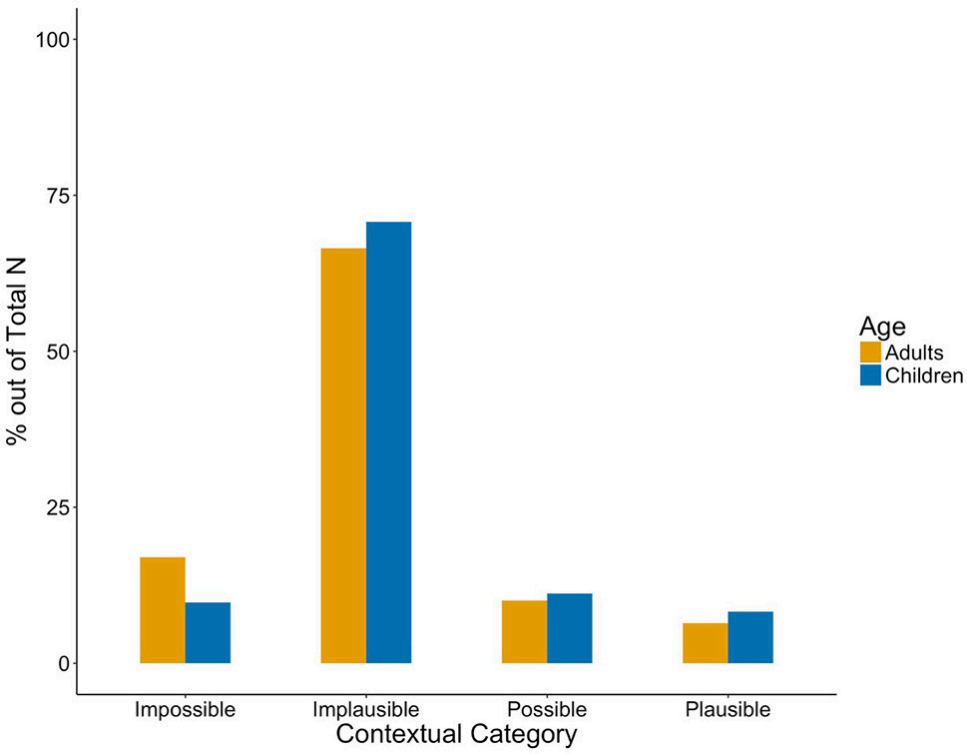
Contextual categories in caregivers' (yellow) and children's (blue) production. Percentages are in proportion to Included utterances per group.

Children's production and mothers' child-directed speech did not differ significantly from each other in either structural or contextual categories (Mann Whitney *U, ps* > 0.1, Kilgarriff, 2001), indicating similar usage patterns across groups (see Figures 2 and 3). Thus, implicature production was similarly low. Even when pooling *Implicature Possible* and *Implicature Plausible* utterances, only 16.49% of adults' and 19.46% children's uses of *some* in the *Included* categories potentially carry an implicature (cf. Degen, 2015, for similarly low rates).

**Figure 4:**
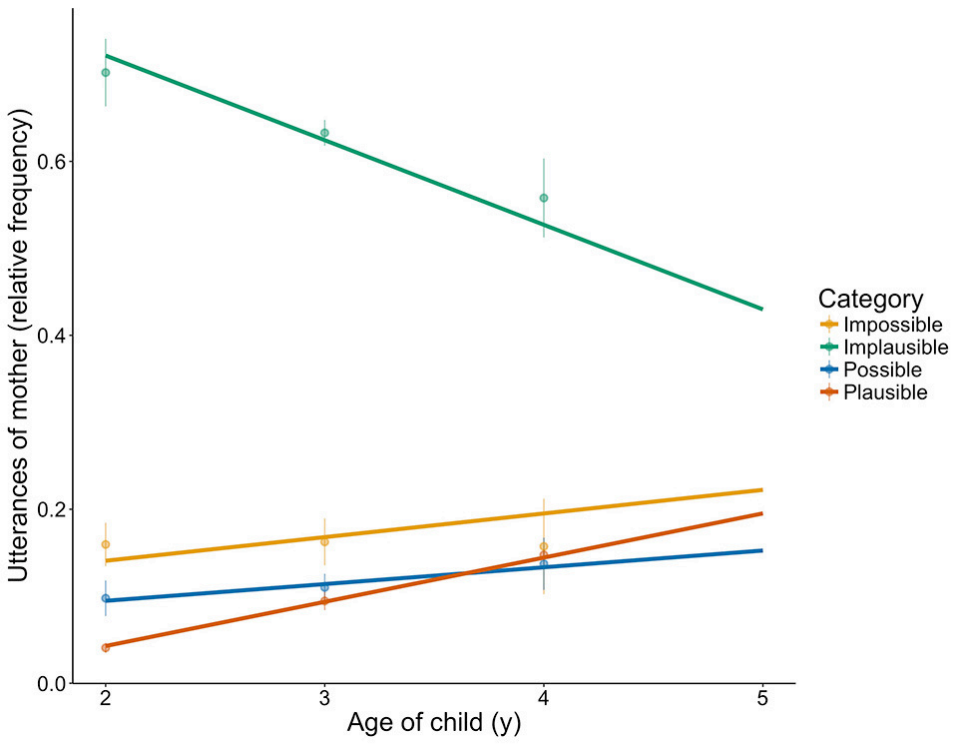
Relative change in the frequency of utterances of the mothers across the childrens age span in the contextual categories *Impossible, Implausible, Possible*, and *Plausible*. The lines reflect the fitted model of the GLMM including Contextual Category and Age, as well as their interaction. res = lmer(Utterances ~ Category^*^Age + (1 + Age | Child); data = d2; REML = F; control = contr).

Interestingly, mothers' usage of *some* changed as a function of the child's age. To analyse how the mothers' implicature production changes, we further coded roughly 300 utterances of the mother after each birthday of her child. To model the data, we fitted a generalized linear mixed model using lme4s lmer function (Bates et al., 2015) with Gaussian error structure and identity link function in R (R Core Team, 2016). Contextual Category and the child's age, and their interaction were included as fixed effects of interest. We also included Child as a random factor to allow for random slopes across participants. The number of utterances in each category at each age was transformed to percentages to standardize the dependent measure across mothers and time points. A reduced model was fit that did not include Contextual Category. A comparison between the reduced model and the full model then allows for conclusions about differential effects in the different contextual categories across the ages. Results can be seen in Table 10 and Figure 4.

**Table 10:** Generalized Linear Mixed Model testing the relative change in the frequency of utterances of the mothers across the childrens ages in the contextual categories *Impossible, Implausible, Possible*, and *Plausible*. res = lmer(Utterances ~ Category^*^Age + (1 + Age | Child); data = d2; REML = F; control = contr).

		**Estimates**	**SE**	**Lower CL**	**Upper CL**	**χ2**	***p***
Full model^(1)^	(Intercept)	0.09	0.05	–0.01	0.18	^(3)^	^(3)^
	Cat: Implausible	0.83	0.07	0.69	0.96	^(3)^	^(3)^
	Cat: Possible	–0.03	0.07	–0.16	0.12	^(3)^	^(3)^
	Cat: Plausible	–0.15	0.07	–0.28	–0.01	^(3)^	^(3)^
	Age	0.03	0.01	–0.00	0.06	^(3)^	^(3)^
	Cat: Implausible:Age	–0.12	0.02	–0.16	–0.08	^(3)^	^(3)^
	Cat: Possible:Age	–0.01	0.02	–0.05	0.03	^(3)^	^(3)^
	Cat: Plausible:Age	0.02	0.02	–0.02	0.07	^(3)^	^(3)^
Impossible^(2)^	(Intercept)	0.17	0.04	0.09	0.25	^(3)^	^(3)^
	Age	–0.00	0.02	–0.04	0.03	0.02	0.89
Implausible^(2)^	(Intercept)	0.87	0.07	0.73	1.00	^(3)^	^(3)^
	Age	–0.08	0.02	–0.12	–0.03	6.09	0.01
Possible^(2)^	(Intercept)	0.05	0.03	−0.02	0.13	^(3)^	^(3)^
	Age	0.02	0.01	–0.00	0.04	3.40	0.07
Plausible^(2)^	(Intercept)	–0.06	0.02	–0.09	–0.03	^(3)^	^(3)^
	Age	0.05	0.00	0.04	0.06	29.67	<0.001

(1) * df = 3*.

(2) * df = 1*.

(3) * Not shown because of having a very limited interpretation as this value is only in relation to the reference level*.

Comparing the full with the reduced model revealed that Contextual Category significantly improved the model fit (χ2 = 150.47, df = 6, *p* < 0.001). Using drop1, the model revealed a significant interaction of Contextual Category^*^Age (χ2 = 39.22, df = 3, *p* < 0.001), suggesting differences between contextual categories at different ages. To analyse these effects further, we split the data according to the different contextual categories. In the model examining the data from the contextual category Impossible alone (*Impossible* split model), there was no significant effect of age. For the *Implausible* split model, the effect of age was significant (χ2 = 6.09, df = 1, *p* = 0.014). For the *Possible* split model, the effect of age only tended toward significance (χ2 = 3.4, df = 1, *p* = 0.065). For the *Plausible* split model, the effect of age was significant (χ2 = 29.67, df = 1, *p* < 0.001). Thus, with each birthday of the child, the mother's number of *Implicature Plausible* instances increased, and the number of *Implicature Implausible* ones decreased. However, neither *Implicature Impossible* nor *Implicature Possible* utterances changed significantly in number across the ages.

### 3.4. Further observations

Before we turn to the possible implications of these results, we would like to present a few additional qualitative observations. These are potentially very interesting and would deserve a systematic investigation that goes beyond the scope of the current study. First, we highlight some cases where children contrast directly the quantifier *some* with other relevant quantifiers. Second, we discuss how modification and *some* in subject position might interact with each other. Finally, we present a few cases where children's utterances were erroneous.

In order to assess how competent young children are with scalar implicatures linked to *some* it is worth looking at whether they spontaneously contrast *some* with other quantifiers on the same semantic “scale.” We found some cases in the corpus where children contrast *some* directly either with *all* or with other quantifiers. Below are four such examples from Thomas and Fraser between 2;02 and 3;08. Further examples can be found in the Supplementary Material.

1. Contrasting *some* with *all* (Fraser, 3;00)
      ^*^FAT: Put all these pieces away.
      CHI: You don't put all of them away.
      FAT: Why?
      CHI: Just do [/] just do (.) some at the time.
      CHI: Not all of them.
      FAT: Not all of them?
      CHI: No.
2. Contrasting *some* with *all* (Mum of Fraser, 2;02)
      ^*^MOT: you dropped *some* pennies.
      CHI: *all* my pennies.
3. Contrasting *some* with *lots* (Thomas, 3;08)
      ^*^CHI: just put some things back in the box.
      INV: do you want to put them back in the box?
      CHI: yes.
      CHI: *some of them* but *not lots of them*.
      INV: okay.
      INV: are you keeping [//] are you keeping everything
      tidy?
      INV: yeah?
4. Contrasting *some* with *some* (Fraser, 3;00)
      ^*^FAT: Yeah.
      CHI: But some girls don't.
      FAT: No.
      CHI: But some girls do.
      FAT: ^*^chuckles^*^ Some boys don't like milk either.
      CHI: But, but…
      FAT: Makes them poorly.

Moreover, we observe a structural hierarchy of implicature plausibility. Indeed, in both adults and children *some* in subject position supported implicature readings, while modifications hindered implicature readings. While these general lines are very clear, the combination of different factors results in a more complex picture. The combination of modification and the *some* phrase in subject position reduces the implicature likelihood (e.g., “Some blue blocks are missing” in the context of many blocks. See also Example 6). In contrast, the combination of modification and the *some of* partitive phrase in subject position makes implicature readings more likely again (e.g., “Some of the blue blocks are missing” in the context of many blocks. See also Example 7). *Some of* highlights the partitive interpretation (84.95% in children), and thus, this structure might serve as a cue to implicature interpretation that outweighs modification; even though the phrase *some of* is not sufficient for an implicature interpretation in and of itself (See Example 8, and see also Degen and Tanenhaus, 2011). Of course, these observations are based on few utterances and more detailed exploration is needed.

(5) Modification (Helen, 4;00)
      ^*^MOT: that's to put that plant in, isn't it?
      CHI: oh yeah.
      CHI: but the plant comes out.
      CHI: I've got *some* even better funny ones.
(6) Modification in subject position hindering implicature
      (Helen, 4;08)
      ^*^CHI: and *some new people* are coming.
      MOT: are they?
      CHI: yeah some new school children that go
      to Wwww_Mwww [% school].
      MOT: right.
(7) *Some of* in subject position overriding modification
      (Mother of Thomas, 2;03)
      ^*^MOT: *some* of the little bubble bath tab eh [//] bubble
      tabs that we've bought haven't been very good, but this
      one is special. Teletubby double bubble it's called.
(8) Uncertain *some of* case (Gina, 4;01)
      ^*^CHI: I wanna touch *some of* this.
      CHI: I wanna touch someone with this.
      CHI: I wanna touch some of this.
      MOT: no it's bacon.

A final observation concerns the type of errors children produced. For all children, the category *Singular NP* seemed to be used erroneously: they used *some* as a determiner with count nouns (e.g., some garden). This resembles a mass noun construction, but would usually be expressed with a simple determiner such as *a*, as we can see in Example 9. This might indicate an overgeneralisation of the frequent *count as mass noun* pattern.

(9) Erroneous *Singular NP* utterance (Eleanor, 2;04)
      ^*^CHI: I've got *some* garden.
      ^*^MOT: you've got a garden?
      CHI: yeah.
      MOT: I like gardens.

Another type of mistake was the production of multiple quantifiers in a row, such as in Example 10.

(10) Several quantifiers (Thomas, 3;01)
      ^*^CHI: I want that's lots *some* few things here.
      MOT: oh alright.
      MOT: you want to look at those books up there?

These cases are mainly present around age 3, when children seem to have acquired the basics of the adult system (Lieven and Behrens, 2012). This pattern of error is particularly interesting and could enlighten our understanding of the development of language structure. In particular, a closer look to these cases could have an impact on syntax-based approaches to scalars (e.g., Chierchia, 2004, 2006), which we discuss briefly below.

## 4. Discussion

In this study we investigated young children's implicature production by looking at the production of *some* in five young children and their mothers. Overall, 14,306 utterances containing *some* were extracted from dense corpora of five British English children aged 2;00 to 5;01 (*N* = 5,276) and alongside that, an equivalent portion for their parents was analysed (*N* = 9,030). All instances of *some* were categorised according to mutually exclusive structural and contextual categories. Structural categories were based on syntactic form while contextual categories considered the contextual environment of the utterance and allowed for judgments on the probability of a scalar implicature being intended.

Analysis of the parents' production revealed that few uses of *some* could be meant to carry an implicature. Our highest implicature plausibility category (*Implicature Plausible*) represents 6.42% of the adult data (8.29% of the children data). A generous approximation of potential intended implicatures pooling together the *Implicature Possible* and the *Implicature Plausible* categories gathers 16.5% of the adult *some* cases (19.5% of the children's). Importantly, the adult results also imply that children are rarely confronted with upper-bound *some*.

Interestingly, the parents' implicature production increased as a function of the children's age. We note an increase of the *Implicature Plausible* cases and a decrease of the *Implicature Implausible* instances over the years. This might be due in part to the large number of *Implicature Implausible* utterances related to food (i.e., “Want some banana”), while *Implicature Plausible* cases highlight a contrast. The change, then, might be brought about by conversations evolving from a focus on more basic desires, such as nutrition, to more complex arguments about variations in the world (“Some girls have brown hair”). While this aspect of our findings would need to be investigated further in future research, the changes in parents' production suggest an evolving learning environment for the child.

The low frequency of implicatures in child-directed speech corroborates the findings of Degen (2015) and Sun (2017) in other adult corpora. Unfortunately, because of differences in methodology our data are not directly comparable to theirs. Degen and Sun both relied on on-line participants ratings on a seven point Likert scale to assess the likelihood of an implicature being intended, while we assessed implicature plausibility according to coding on a four categories scheme performed by one or two coders. The proportion of combined *Implicature Possible* and *Implicature Plausible* cases we find (16.5%, for two out of four categories) is lower than that of the ratings higher than midpoint in either studies (44.7% for Degen and 64% for Sun). Note that Sun's is already higher than Degen's and that the short study by Huang and Snedeker (2009b) reports that a relatively high 42% *some* occurrences “unambiguously referred to a subset” (Huang and Snedeker, 2009b, p. 410). It is unclear that looking at midpoint ratings is the best way to compare these different data sets. For instance, Degen finds that only 14.7% (Degen, 2015, p. 12) of her data corresponds to the highest ratings while, under what she considers to be the best analysis of the components, 28% are generated by an upper-bound interpretation (Degen, 2015, p. 16). Yet, even from this angle, our data seem to foster less upper-bound *some* instances than these other studies.

The discrepancy in the various findings might stem from two sources: differences in the nature of the corpora, on the one hand, and differences in the way implicature plausibility was established on the other. First, corpora varied greatly in kind and in size: we coded 4,172 *included some* instances taken from child-directed speech in every day activities, while Huang and Snedeker (2009b) looked at 50 occurrences of *some* from the British National Corpus, Degen (2015) analysed 1,748 from telephone dialogues and 200 cases taken from tweets were rated in Sun (2017). This diversity might influence *some* distributions. For instance, Sun (2017, p. 80) notes that a higher percentage of partitive *some* in her corpus might, in part, explain why she finds higher implicature plausibility ratings than Degen (2015). It is also possible that parents addressing a young child intend less upper-bound readings of *some*. Such an interpretation fits well with our finding that parent *Implicature Plausible* instances increase as their children grow. We found many utterances of the “want some grapes” type in child-parent interactions; probably substantially more than we would in adult conversation. Yet, without further evidence, this conclusion is premature since several other parameters might explain a somewhat lower frequency of upper-bound *some* cases in our data.

Second, diverging findings might come down to differences in data collection (rating vs. coding), implicature assessment tests (existence of a subset vs. *not all* paraphrases) or exclusion criteria for irrelevant cases. For example, Sun (2017) filtered out occurrences falling under the scope of negation, in questions or conditional antecedents, and Degen (2015) took out singular *some* cases, while we did neither. The crucial parameter in explaining the difference between our results and those of Degen and Sun is probably how implicature plausibility was coded for. Indeed, untrained Mechanical-Turkers are likely to be more lenient in their assessment than linguistically trained coders instructed to be conservative when granting implicature plausibility (to prevent overestimating implicature production in toddlers). Importantly, discussion about differences in findings and methods of assessments should not distract us from the striking convergence of all available adult corpus studies on a low proportion of upper-bound interpretation for *some*.

The relatively low frequency of adult implicature production found in all four corpora clearly speaks against what Degen (2015) coined the Frequency Assumption. No matter how one looks at the data it is impossible to claim that the predominant reading of *some* is prone to implicature. This important, and now robust, finding is difficult to reconcile with theories assuming that *some* commonly induces implicatures, such as syntactic accounts (e.g., Chierchia et al., 2012) or Horn's (1984, 1989) Generalised Conversational Implicature thesis and Levinson's default theory (Levinson, 2000), which maintains that *some* will give rise to a scalar implicature by default, unless the context blocks the inference. Additionally, as Degen (2015) argues, the low frequency of *some*-related implicatures in corpus research also has consequences for the so-called Literal-First-Hypothesis (Huang and Snedeker, 2009a). According to this thesis, the interpretation of upper-bound *some* follows a two-stage processing model where it always appears with a delay, after the lower-bound reading has been computed. This hypothesis is not directly contradicted by the low frequency of upper-bound readings in corpora, but it makes it more difficult to test. Indeed, while several researchers have shown that deriving a scalar implicature linked to *some* comes at a cognitive cost and is processed slower (Breheny et al., 2006; De Neys and Schaeken, 2007; Huang and Snedeker, 2009a, 2011; Degen and Tanenhaus, 2011; Bergen and Grodner, 2012; Bott et al., 2012), this could be due to the low frequency of the reading rather than to a two-stage processing. After all, as Degen (2015) points out, frequency is a well-established factor in psycholinguistics and there is no reason to assume it would not influence pragmatic aspects, too.

Interestingly, structural elements influence implicature plausibility both in the production by parents and children. Here, too, our data corroborates the work of Degen (2015). For instance, *some* in the subject position increases the likelihood of an implicature (as in Degen, 2015, p. 28). In contrast, modification reduces implicature likelihood; although, this finding is not as pronounced in Degen's analysis (Degen, 2015, p. 29). Additionally, some structural categories seemed related to implicature plausibility. *Singular Some* cases, for instance, did not include any *Implicature Plausible* cases and indeed they were part of the excluded categories in Degen (2015). On the other hand, the majority of *Some Of* cases did support an implicature reading; as was found both by Degen (2015, p. 23) and Sun (2017, p. 80). While partitive *some* does not always promote an implicature, it often does and more often so than non-partitive *some* (see also Degen and Tanenhaus, 2011).

It is worth noting that we found a high proportion of *some* uses in constructions typical of English (as opposed to many other Indo-European languages) and where *some* cannot necessarily be linked to implicature production. Specifically, the determiner *some* is frequent in English (e.g., “I need some batteries”). While it might be meant to carry an implicature when a set of batteries is present, it can also be a simple determiner phrase when no set is referenced (28.22% of all utterances in adults and 20.41% of all utterances in children, see also Bagassi et al., 2009; Degen, 2015, for a thorough discussion). This reading is widespread in English, but would be conveyed without recourse to the quantifier *some* in other languages (see Supplementary Material for examples and their translations). Such instances were categorised as *Implicature Implausible* and might induce a lower rate of implicature plausibility than in other languages. In her work, Degen (2015) concludes that implicatures are highly dependent on syntactic, semantic, and pragmatic influences from the context and appear to be probabilistic in nature—i.e., rather than being an all-or-nothing phenomenon it makes sense to ask to what degree they arise (see also Degen and Tanenhaus, 2015). Our results support her argument: in the present study, implicature-compatible utterances in both child-directed speech and children's production are low in frequency, but seem dependent on syntactic and contextual information.

The most surprising aspect of the data, of course, is that children produce *Implicature Plausible* instances of *some* very early on and at rates matching those of their parents. The children's production of *some* mirrors that of their parents' in all aspects. Although, this has also been found for other structural phenomena in language (e.g., Kidd et al., 2007), the degree of resemblance between adult and child production both in the structural and in the contextual categories is remarkable (see Figures 2 and 3). The overall pattern of the findings suggests children master the use of *some* early on with a distribution of *some* mimicking child-directed speech. This is what one would expect considering work on frequency matching between parents' and children's speech (Ambridge et al., 2015). It seems natural that the children use *some* highly frequently in non-implicature, more low scope formulaic utterances such as “I want some banana,” since parents use these constructions very frequently. The real surprise, then, is that children produce scalar implicatures, which are regarded as a complex pragmatic inference, so early. Although parents' production suggests children are rarely confronted with instances of *some* meant to carry implicatures, utterances favouring a lower-bound interpretation nonetheless appear in their third year of life (or were present as soon as the recording started), shortly after their first production of *some* (Eleanor 2;04;02, Fraser 2;03;06, Thomas 2;09;11, Gina 3;00;04, Helen 3;00;10; see Table 2). As for their parents, *some* is produced in many different syntactic structures; implicatures appear to be rare and dependent on linguistic structure and context. Nevertheless, almost as soon as they acquire *some*, we see the children producing it competently, including upper-bound uses.

How can we account for such an early production of implicatures? There is ample evidence that children calculate intentions in communicative contexts even preverbally (e.g., Tomasello, 2008). Indeed, much work, in language acquisition also suggests that they could not learn to speak without impressive pragmatic abilities (e.g., Bloom, 2000; Tomasello, 2003; Clark, 2016). Once they have figured out the semantics of *some*, children might therefore be able to work out how to produce the implicature. An additional element is necessary, of course, the understanding that *some* might be on a semantic scale with other quantifiers (*all, many, most*), or at least that its meaning can contrast with theirs. Examples (1)-(3) above indicate they do so early on. Yet, such an interpretation of early scalar implicature production and, more generally, our findings contrast with work showing that *some*-related implicatures are understood relatively late in childhood, and thus, call for an explanation. On the one hand, our production results corroborate the study by Katsos and Smith (2010) suggesting that implicature production arises early. On the other hand, the earliest children have been found to understand *some*-related scalar implicatures is 4 (Pouscoulous et al., 2007; Katsos and Bishop, 2011), while our findings suggest that they can produce *some* with an upper-bound reading from the age of two. The gap between these two sets of evidence must be bridged.

An account along lexicalist lines (e.g., Barner et al., 2011) might find it difficult to contend with such early implicature production. If toddlers have not associated *some* with its lexical scale (*many, most, all*), this should affect their ability to produce, as well as comprehend, implicatures. Importantly, examples where children's use of *some* is directly contrasted with another member of the semantic scale (*all* or other, see Examples 1 - 4 and Supplementary Material), reinforce a picture where children master the contrast set of *some* from a very early age—as young as 2;03 for some of them. These cases indicate that the *Implicature Plausible* instances found in child production are not merely an artifact of our way of categorising *some*-utterances, but truly reflect the ability of very young children to intend scalar implicatures linked to *some*. They also speak further against a lexicalist account of scalar implicature acquisition. Therefore, an approach on the development of scalars integrating several contextual factors might be more appropriate to reconcile the experimental comprehension findings with our production data.

Several elements may explain children's behaviour in comprehension experiments such as their pragmatic tolerance (Katsos and Bishop, 2011), the relevance of the implicature in context (Papafragou and Musolino, 2003; Guasti et al., 2005; Skordos and Papafragou, 2016), and children's limited processing resources when faced with an infrequent, relatively effortful inference (Reinhart, 2004; Pouscoulous et al., 2007). Indeed, pragmatic tolerance constrains experimental measures of implicature comprehension, since children might be inclined to judge a sentence as correct despite pragmatic infelicity. But, of course, pragmatic tolerance would have no impact on production. Similarly, while implicature comprehension might be affected by how relevant the scalar implicature is in context, relevance does not influence production: if a speaker intends to produce an implicature, then it is *a priori* relevant to them. These factors combined with children's limited exposure to *some*-related implicatures may be sufficient to account for the discrepancy between production and comprehension. In this view, children are capable of producing and inferring *some*-related implicatures from their third year of life and any difficulty in understanding them in experimental settings is to be attributed to factors outside their semantic and pragmatic competence.

This type of account also resonates with experimental findings suggesting a much earlier comprehension of linguistic pragmatic phenomena than previously thought. Indeed, while preschoolers find most pragmatic inferences challenging on traditional metalinguistic tasks such as explaining or judging the truth value of an utterance, a few recent studies indicate that they fare much better with paradigms using act-out or picture selection tasks: 3-year-olds understand other pragmatic phenomena (e.g., Berger and Höhle 2012 on presupposition; Falkum et al. (2017) on metonymy; Pearson (1990) on metaphor), but also other implicatures (Schulze et al., 2013, on relevance implicatures) and even other types of scalar implicatures (Stiller et al., 2014, on *ad hoc* scalar implicatures).

In the past decade a lot of work has been devoted to children's comprehension of *some*. In fact, our knowledge of implicature acquisition is largely based on their understanding of this one expression. A systematic corpus analysis of how toddlers hear and produce it should therefore be essential to any informed argument in the debate. The findings indicate that children begin producing and interpreting implicatures in a pragmatic way during their third year of life, very soon after they first produce *some*. Thus, almost as soon as they acquire *some*, children produce it competently and mirror adult behaviour. Their production of *some* implicatures is low but matches their parent's input in frequency. In both children and adults *some* appears to be multifaceted and implicatures are infrequent, and both structurally contextually constrained. Our findings add to a growing body of evidence showing that the upper-bound reading of *some* is much less frequent in adult speech than some scholars would have had us believe. Our study is also the first to go against the popular belief in some psychology and linguistics circles that children do not produce implicatures, much less so lexicalized scalar implicatures, at an early age. Yet, it does by no means answer all the questions. The method we used has its flaws in that it relies on coder judgment; it has its strengths, too, in the nature and size of the corpus we used. The similarity between other adult findings and ours, and the striking resemblance between our adult and children results give us reasonable confidence in the soundness of our paradigm. In any case, this work should be expanded by experimental research looking at children's production of *some* and other implicatures. An important question which still requires a more fine-tuned answer – both empirically and theoretically – is how children can appear to fare so poorly with implicatures in experimental paradigms if the basic mechanisms are in place so early.

## Data availability statement

A file including data and coding for this study can be found on the Open Science Framework at osf.io/g6psr.

## Author contributions

NP, EL, and SE designed the study. EL provided the dataset. SE coded and analysed the data; SE, NP, and EL wrote the manuscript. All authors discussed the results and commented on the manuscript.

### Conflict of interest statement

The authors declare that the research was conducted in the absence of any commercial or financial relationships that could be construed as a potential conflict of interest.
