# Supplementary material for: Some Pieces Are Missing: Implicature Production in Children
Source: Front Psychol. 2018 Oct 24;9:1928. doi: 10.3389/fpsyg.2018.01928 (PMC6207867; doi:10.3389/fpsyg.2018.01928)
Supplement: Supplementary file 1 [file Data_Sheet_1.pdf]

Supplementary Material: Some pieces are missing: Implicature production in children

1 CONTRASTING *SOME* IN THE CORPUS

(1) Contrasting *some* with *all* (Helen, 3;00)
*MOT: There's all this stuff that I'm trying to sort out and it doesn't help if you're jumping all over them, does it?
CHI: I [*] not jumping.
CHI: But, but - but - er - I must get out now.
CHI: Some things.
MOT: Pardon ?
CHI: I must get out with...
CHI: ...all my things.

(2) Contrasting *some* with *all* (Fraser, 3;00)
*CHI: All finish.
MOT: We've got two that are all finished, haven't we?
CHI: Yeah.
CHI: But those ones, they're missing some pieces.
MOT: They are, aren't they?
CHI: Those ones are missing some pieces.
MOT: Never mind.

(3) Contrasting *some* with *all* (Gina, 4;03)
*MOT: So am I allowed to get in this bath with you?
CHI: Yeah!
MOT: Is there room with all those toys in it?
CHI: Well I'll take out - some toys away.
CHI: *Humming/babbling*.
CHI: *Humming/babbling*.
CHI: Right come on, Mum.

(4) Contrasting *some* with *nobody* (Helen, 4;05)
*MOT: Does she?
MOT: But did nobody take you to the teacher?
MOT: Did nobody tell the teacher that you'd fallen over?
CHI: Some - some people did.
MOT: Oh.
MOT: Who took you in?
CHI: I don't know.

(5) *Some Of* phrase (Helen, 4;05)
*CHI: No.
MOT: Has she been growing in your tummy?
CHI: Yeah.
CHI: When some babies when they're growing in their mummy's tummy sometimes some of them get scared.
MOT: Yeah.
MOT: *tuts*.
MOT: (Be)cause they've not seen us before.

2 THE QUANTIFIER *SOME* IN OTHER LANGUAGES

(6) English
I would like some tea, please.

(7) French
Je voudrais du/*certains/*quelques thé.
I would like of-the/*some tea.

(8) Spanish
Quisiera un poco de/*algunos te.
Want a bit of/*some tea.

(9) Italian
Vorrei un po' di/ *alcune te.
Want a bit of/ *some tea.

(10) German
Ich hätte gerne etwas/ *einige/ *ein paar Tee.
I would like a bit/ *some/ *a few tea.

(11) Dutch
Ik zou graag wat/*enkele thee hebben.
I would like a bit/*some tea have.

(12) Russian
Ya khotel by/*nekotoryy chayu.
I would like /*some tea.

(13) Finnish
Haluaisin teetä.
Would like *some tea.

(14) Georgian
minda *zogiert'i ch'ai.
want *some tea.

(15) Estonian
Ma tahaksin *mõnesid/*natuke teed.
I would like *some tea.
